# Identification of miRNAs in extracellular vesicles as potential diagnostic markers for pediatric epilepsy and drug-resistant epilepsy via bioinformatics analysis

**DOI:** 10.3389/fped.2023.1199780

**Published:** 2023-07-03

**Authors:** Yucai Ruan, Xuhui Deng, Jun Liu, Xiaobing Xiao, Zhi Yang

**Affiliations:** ^1^ Department of Pediatrics, Yue Bei People’s Hospital, Shantou University Medical College, Shaoguan, China; ^2^ Department of Neurology, Yue Bei People’s Hospital, Shantou University Medical College, Shaoguan, China; ^3^ Medical Research Center and Clinical Laboratory Medicine, Yue Bei People’s Hospital, Shantou University Medical College, Shaoguan, China

**Keywords:** extracellular vesicles, pediatric epilepsy, miRNAs, drug-resistant epilepsy, biomarkers

## Abstract

**Background:**

Pediatric epilepsy (PE) is a common neurological disease. However, many challenges regarding the clinical diagnosis and treatment of PE and drug-resistant epilepsy (DRE) remain unsettled. Our study aimed to identify potential miRNA biomarkers in children with epilepsy and drug-resistant epilepsy by scrutinizing differential miRNA expression profiles.

**Methods:**

In this study, miRNA expression profiles in plasma extracellular vesicles (EV) of normal controls, children with drug-effective epilepsy (DEE), and children with DRE were obtained. In addition, differential analysis, transcription factor (TF) enrichment analysis, Gene ontology (GO) analysis and Kyoto Encyclopedia of Genes and Genomes (KEGG) enrichment analyses, and target gene prediction were used to identify specifically expressed miRNAs and their potential mechanisms of action. Potential diagnostic markers for DRE were identified using machine learning algorithms, and their diagnostic efficiency was assessed by the receiver operating characteristic curve (ROC).

**Results:**

The hsa-miR-1307-3p, hsa-miR-196a-5p, hsa-miR-199a-3p, and hsa-miR-21-5p were identified as diagnostic markers for PE, with values of area under curve (AUC) 0.780, 0.840, 0.832, and 0.816, respectively. In addition, the logistic regression model incorporating these four miRNAs had an AUC value of 0.940, and its target gene enrichment analysis highlighted that these miRNAs were primarily enriched in the PI3K-Akt, MAPK signaling pathways, and cell cycle. Furthermore, hsa-miR-99a-5p, hsa-miR-532-5p, hsa-miR-181d-5p, and hsa-miR-181a-5p showed good performance in differentiating children with DRE from those with DEE, with AUC values of 0.737 (0.534–0.940), 0.737 (0.523–0.952), 0.788 (0.592–0.985), and 0.788 (0.603–0.974), respectively.

**Conclusion:**

This study characterized the expression profile of miRNAs in plasma EVs of children with epilepsy and identified miRNAs that can be used for the diagnosis of DRE.

## 1. Introduction

Epilepsy is a common, recurrent neurological disease in children, affecting their brain development and threatening their physical and mental health (1). According to statistics, pediatric epilepsy (PE) affects the health of 65 million children worldwide. The prevalence of PE ranges from 3.2/1,000 to 6.3/1,000 in developed countries and 5/1,000 in China (1–3). PE primarily affects children under the age of seven, with the highest incidence occurring within the first year of life (4). Children with epilepsy are less able to express themselves and have atypical clinical symptoms at this age. During this critical period of brain development, epilepsy that is not diagnosed early and treated with timely interventions will cause brain damage and severe and lasting neuropsychiatric disease (5). Currently, the early diagnosis of epilepsy relies on electroencephalogram and imaging during the seizure period. However, repeated and multiple monitoring is required to confirm the diagnosis. Moreover, the pathogenesis of epilepsy remains unclear. Therefore, the search for new biological markers that can be used for the diagnosis of PE is crucial. Currently, bioinformatics is widely used in identifying diagnostic and prognostic biomarkers for multiple diseases. Based on bioinformatics, several researchers have analyzed transcriptomic data from public databases and combined them with clinical information to identify prognostic biomarkers for a variety of tumors, providing a new dimension for the elucidation of tumorigenesis and treatment (6–8). Giambò et al. found that pesticide exposure could induce genomic and epigenetic alterations by integrating multiple datasets (9). Symonds et al. suggested that epilepsy genomics plays a critical role in understanding the mechanisms of disease and developing new drug targets (10). Therefore, in this study we also explored the potential pathogenesis of pediatric epilepsy and identified potential diagnostic biomarkers via bioinformatics.

The primary treatment scheme for epilepsy is medication (11). However, 1/3 of patients who fail to fully control their seizures despite treatment with medications alone or a combination of antiepileptic drugs develop drug-resistant epilepsy (DRE) (11, 12). A study in 2018 revealed that 36% of 1,795 patients newly diagnosed with epilepsy developed persistent DRE and were less responsive to medication (13). DRE leads to stunted growth and poor social adaptation in children, along with an increased risk of death and a significant financial strain on society and families. However, there is a lack of understanding of the potential mechanisms underlying the pathogenesis of DRE and the effective follow-up management schemes. In addition, potential biological markers that can be used to predict response to antiepileptic drugs are unavailable. Therefore, there is a need for searching biological markers that can identify DRE at an early stage to facilitate timely changes in treatment strategy and to improve the patients' quality of life.

MicroRNAs (miRNAs) are 19–25 nucleotides, small non-coding RNAs, and are stably present in serum and tissues (14). miRNAs are key regulators of normal physiological functions and are crucially involved in cell growth and apoptosis, as well as tissue development and differentiation (15). MiRNA's mechanisms of action are well established. Mature miRNAs degrade mRNA and reduce protein production primarily via the binding of the 3' region (non-coding) to the 5' region (non-coding) of mRNA (16). Some researchers have indicated that miRNAs are important in the regulation of neurodegenerative lesions and epileptic pathology (17, 18). In recent years, miRNA has also been increasingly studied in various central nervous system (CNS) diseases (19–21). Furthermore, it has shown promising significance in the early diagnosis and therapeutic assessment of Alzheimer's disease, psychiatric disorders, and depression (22–24). However, the function of the miRNAs in the onset and progression of PE is still not clear.

Extracellular vesicle (EV), a vesicular structure wrapped by a cell-secreted lipid bilayer, is highly lipid soluble (25). EVs are rich in miRNAs that can be transmitted between cells (26). EV-derived miRNAs have an important role in the maintenance of normal physiological functions (27). Additionally, EVs with a diameter of 50–150 nm has good biocompatibility, can cross the blood-brain barrier, and are considered good candidates for the diagnosis of CNS disease (28). EVs have been also reported in intercellular communication and their regulation is paramount. Genc et al. reported that glioblastoma multiforme-derived exosomes could cause neuronal damage (29). Wang et al. summarized the potential of exosomes as biomarkers of neurodegenerative lesions and the underlying mechanisms for regulating oxidative stress (30). In addition, several investigators have described the promise of EVs in the diagnosis of epilepsy and as an adjunct to antiepileptic therapy (31, 32). In this research, the characteristic expression profiles of miRNAs in plasma circulating EVs of children with epilepsy and healthy children were obtained from public databases using bioinformatics approaches. In addition, biological markers that can be used to differentiate children with epilepsy and those with DRE were screened, and their potential biological functions were explored.

## 2. Material and methods

### 2.1. Data downloading and processing

In this study, we take “pediatric epilepsy” and “exosomes” as keywords for the retrieval in GEO datasets (https://www.ncbi.nlm.nih.gov/geo/). Our exclusion criteria were (1) non-blood samples and (2) non-mirRNA datasets. Expression data for miRNAs from serum EVs were obtained from the GEO database, GSE193842 (33). This cohort contained 10 healthy controls, 12 children with drug-effective epilepsy (DEE), and 13 children with DRE. The Illumina NovaSeq 6000 platform provided the assay data for this cohort. All patients included in the study underwent a rigorous screening process. Ethical approval from the hospital and informed consent from patients were obtained for this study. Furthermore, the SVA package was utilized to perform background correction and normalization on the gene expression data, ensuring accurate analysis of differential expression across all groups.

### 2.2. Identification of differential expressed miRNAs

The limma package was employed to assess the differentially expressed miRNA (DEmiRNAs) between children with epilepsy and healthy controls and between children with DEE and those with DRE. The Benjamini–Hochberg method was employed to measure the false discovery rate (FDR). FDR < 0.05 and log_2_FC absolute value greater than or equal to 0.5 were used as screening criteria for DEmiRNAs. The ggplot2 package was utilized to present the outcomes of the differential analysis and exhibit them via the volcano plot. The ComplexHeatmap package was utilized for the purpose of visualizing heat maps, processing normalization, and performing Euclidean distance clustering. Conduct an analysis of the distinct and shared components of the data across all groups utilizing the ComplexHeatmap package, generate a visual representation of the findings, and construct a Venn diagram utilizing the ggplot2 package and VeenDiagram package.

### 2.3. Prediction of target gene

TargetScan (http://www.targetscan.org/vert_71/), miRDB (http://mirdb.org/), and miRTarBase (https://mirtarbase.cuhk.edu.cn/) databases predicted the miRNAs potential target genes.

### 2.4. Functional enrichment analyses

The functional enrichment analysis tool (FunRich, http://funrich.org/download) is a web tool employed for gene functional enrichment and interaction network analysis. The FnuRich-based tool analyzed the primary enrichment pathways of differentially expressed miRNAs (DEmiRNAs) and the potential transcription factors (TFs) regulating their expression. Gene ontology (GO) analysis and Kyoto Encyclopedia of Genes and Genomes (KEGG) pathway enrichment analyses were carried out with the R packages “org.Hs.eg.db” and “clusterProfiler”.

### 2.5. Bioinformatics and statistical analyses

STRING (https://cn.string-db.org/) database, which contains rich data on protein-protein interaction (PPI), was used to construct network relationships for interactions between miRNA target genes. In this study, miRNAs that can be used to differentiate DRE were screened by LASSO regression model for dimensionality reduction and a 10-fold cross-validation procedure. In addition, ROC curves were plotted by the pROC package, and their AUC values were calculated. Wilcoxon rank-sum test was employed to analyze the differences in the non-normally distributed variables. *P* < 0.05 signified a remarkable difference.

## 3. Results

### 3.1. Identification of DEmiRNAs in children with epilepsy and normal controls

**Figure 1:**
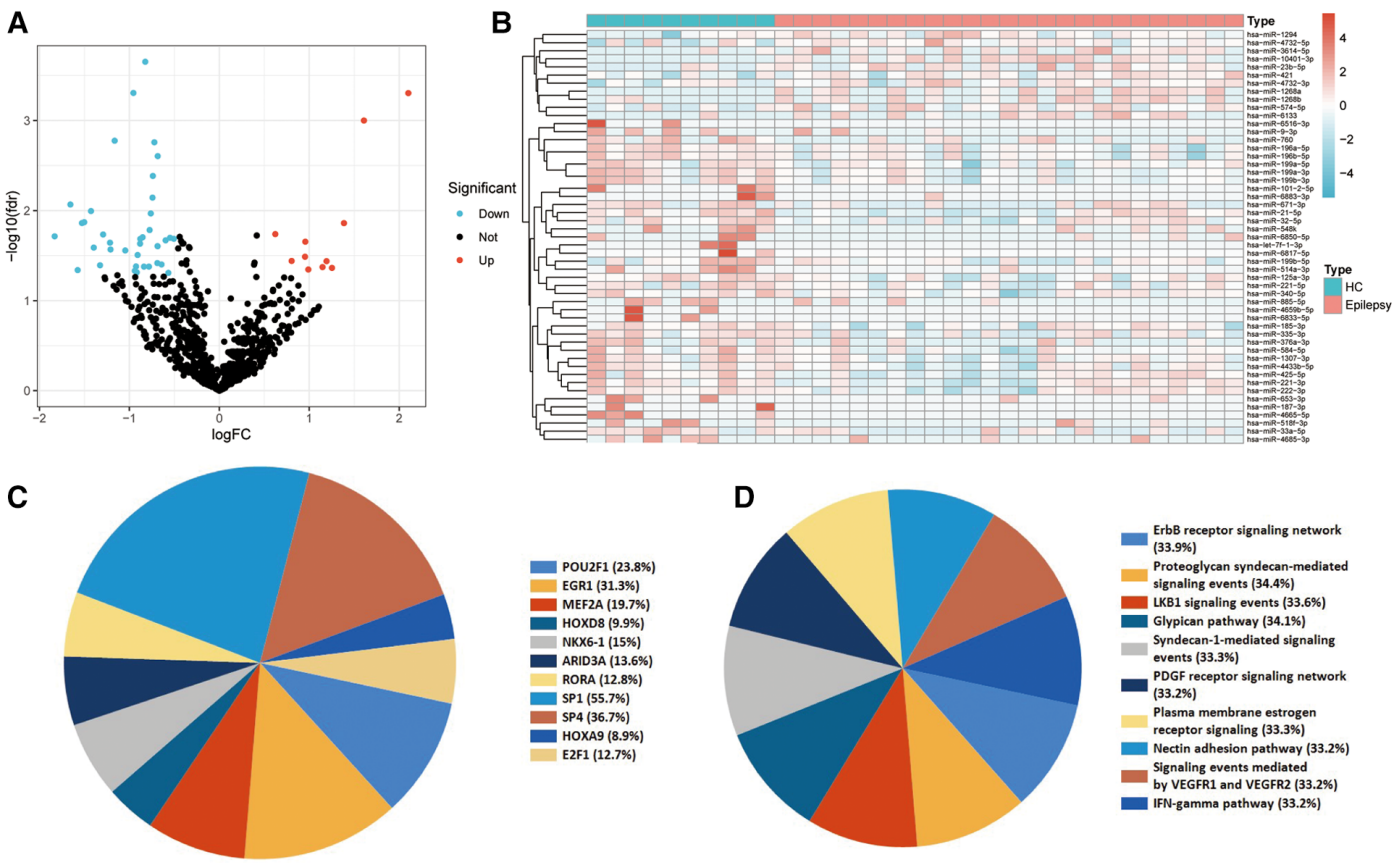
Identification and functional enrichment analyses of the differentially expressed miRNA (DEmiRNAs). (**A**) Differential expression of plasma EV-derived miRNAs between children with epilepsy and normal controls. The blue dots denote miRNAs that have undergone down-regulation, while the red dots indicate miRNAs that have undergone up-regulation. (**B**) Heat map of differential miRNA expression between children with epilepsy and normal controls. (**C**) Transcription factors (TFs) enrichment analysis of DEmiRNAs. TFs exhibiting *P* values below 0.05 were chosen for presentation. (**D**) Pathway enrichment analysis of DEmiRNAs. The top ten enrichment pathways were selected for display.

Initially, miRNA expression was analyzed in plasma exosomes derived from children with epilepsy and normal control children. In total, 11 miRNAs were identified to be up-regulated, and 40 were down-regulated in children with epilepsy (Figure 1A). The expression of DEmiRNAs in the two categories is shown in the heatmap (Figure 1B). TF enrichment analysis revealed that these DEmiRNAs may be regulated by POU2F1, EGR1, MEF2A, HOXD8, NKX6-1, ARID3A, RORA, SP1, SP4, HOXA9, and E2F1 TFs (Figure 1C). In addition, enrichment analysis suggested that these DEmiRNAs were primarily enriched in the IFN-gamma pathway, signaling events mediated by VEGFR1 and VEGFR2, plasma membrane estrogen receptor signaling, and PDGF receptor signaling network (Figure 1D).

### 3.2. Identification of diagnostic biomarkers for PE

**Figure 2:**
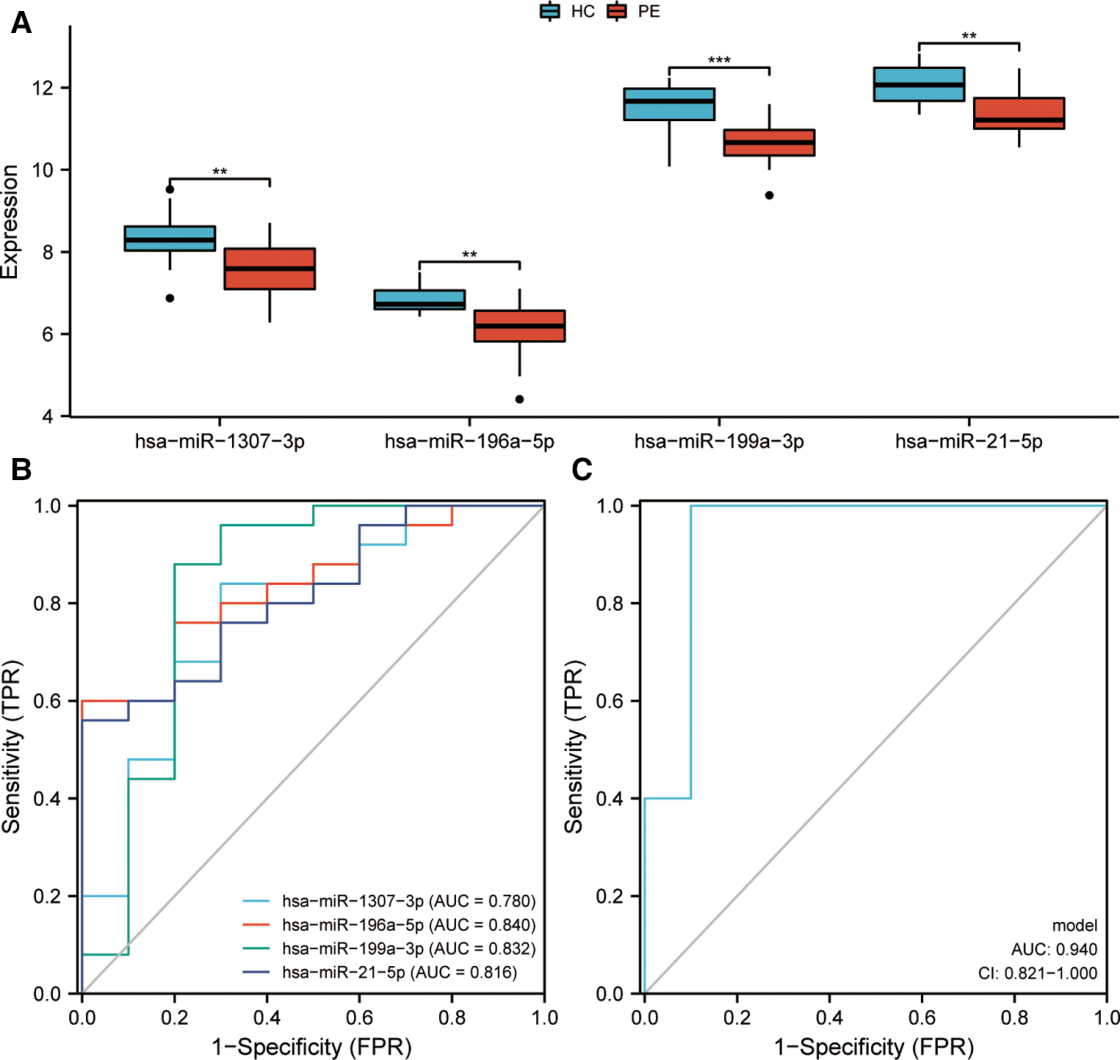
Identification and evaluation of markers for pediatric epilepsy (PE). (**A**) Expression of miRNAs between children with epilepsy and healthy control (HC). (**B**) Receiver Operating Characteristic (ROC) curves assessing the diagnostic efficiency of miRNAs. Area Under Curve, AUC. (**C**) A ROC analysis was conducted to evaluate the diagnostic efficacy of a diagnostic model that was developed through logistic regression. CI, confidence interval.

Initially, genes exhibiting zero expression level and a missing rate exceeding 30% were eliminated. Subsequently, an AUC analysis was conducted on the remaining genes, and the top four miRNAs (hsa-miR-1307-3p, hsa-miR-196a-5p, hsa-miR-199a-3p, and hsa-miR-21-5p) were identified as promising diagnostic biomarkers for PE. In addition, further differential expression analysis highlighted that the levels of expression of these four miRNAs were remarkably higher in healthy controls than in children with epilepsy (Figure 2A). Subsequently, it was shown by these four miRNAs' ROC curves that they all exhibited excellent performance in distinguishing children with epilepsy (hsa-miR-1307-3p: AUC = 0.780; hsa-miR-196a-5p: AUC = 0.840; hsa-miR-199a-3p: AUC = 0.832; and hsa-miR-21-5p: AUC = 0.816) (Figure 2B). A diagnostic model based on these four miRNAs was further constructed by logistic regression. The precise and comprehensive parameters are presented in Supplementary Table S1. ROC analysis revealed that the model had good discrimination power with an AUC value of 0.94 (Figure 2C).

### 3.3. Target gene prediction and enrichment analyses

**Figure 3:**
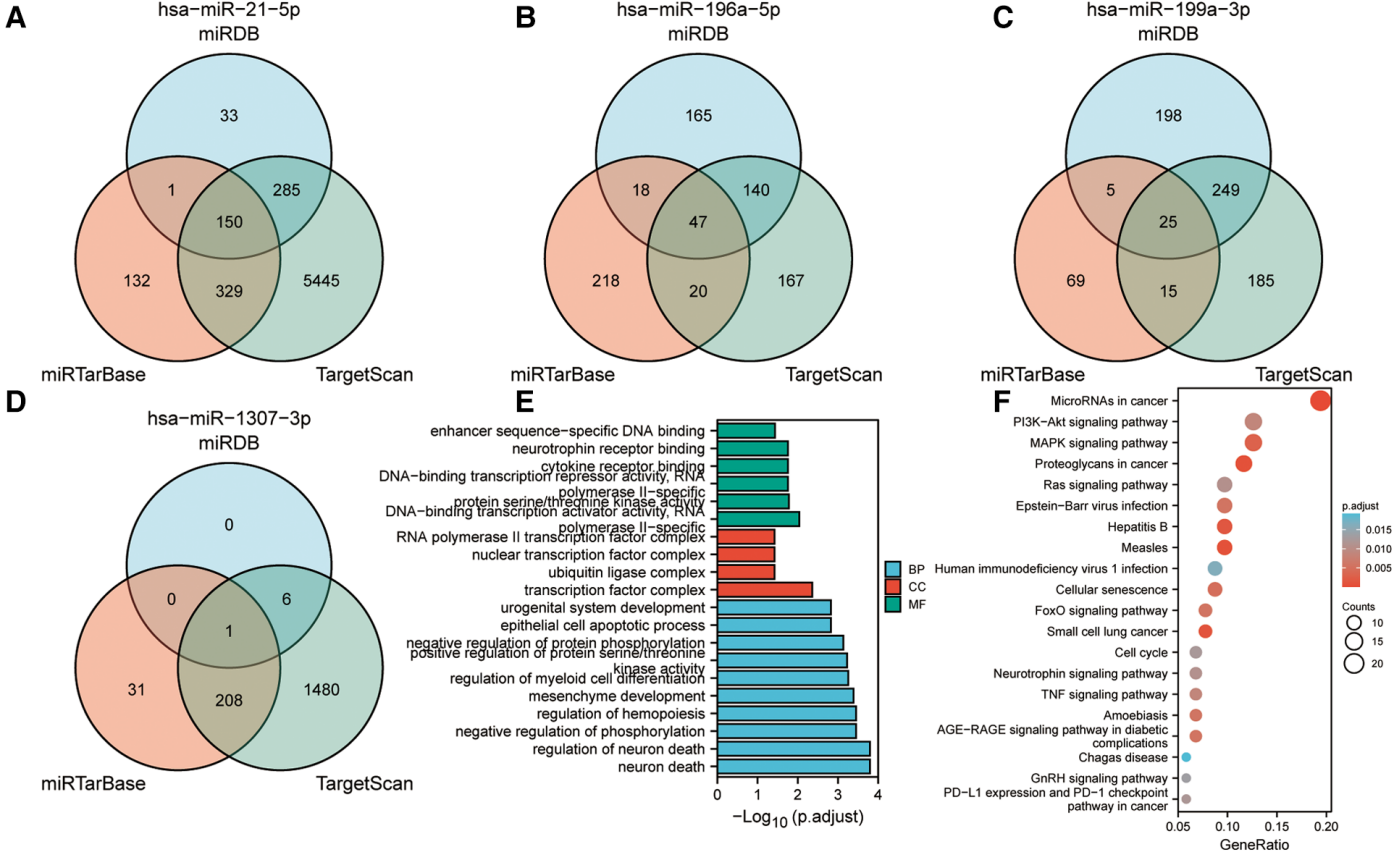
Target gene prediction of miRNA and enrichment analyses. (**A**) Target gene prediction of miR-21-5p, (**B**) miR-196-5p, (**C**) miR-199a-3p, and (**D**) miR-1307-3p. The target genes were predicted using three distinct databases, and the intersection of the predicted target genes across all three databases was visualized through a Venn diagram. (**E**) Gene ontology (GO) enrichment analysis of target genes. (**F**) Kyoto Encyclopedia of Genes and Genomes (KEGG) enrichment analysis of target genes. The presented items of enrichment exhibit a adjust *P* < 0.05.

MiRNAs exert regulatory functions *in vivo* by targeting mRNAs. Three miRNA target gene prediction websites, miRTarBase, TargetScan, and miRDB, were searched for the prediction of the potential target genes of these miRNAs, thus clarifying the possible role of these four miRNAs. Subsequently, the prediction results from these three websites were intersected to increase the predictive robustness. Hsa-miR-21-5p, hsa-miR-196a-5p, hsa-miR-199a-3p, and hsa-miR-1307-3p had 150, 47, 25 and, 1 potential target genes, respectively (Figures 3A–D). GO enrichment analysis highlighted that these target genes were primarily involved in several biological processes including regulation of neuron death, neuron death, regulation of myeloid cell differentiation, and regulation of hemopoiesis. In addition, these target genes, primarily located in the nuclear transcription factor complex, ubiquitin ligase complex, and transcription factor complex, were involved in multiple molecular functions such as DNA-binding transcription activator activity, cytokine receptor binding, and neurotrophin receptor binding. KEGG enrichment analysis highlighted that these target genes were liked to the PI3K-Akt, MAPK, and Ras signaling pathways, and proteoglycans in cancer pathway (Figure 3F).

**Figure 4:**
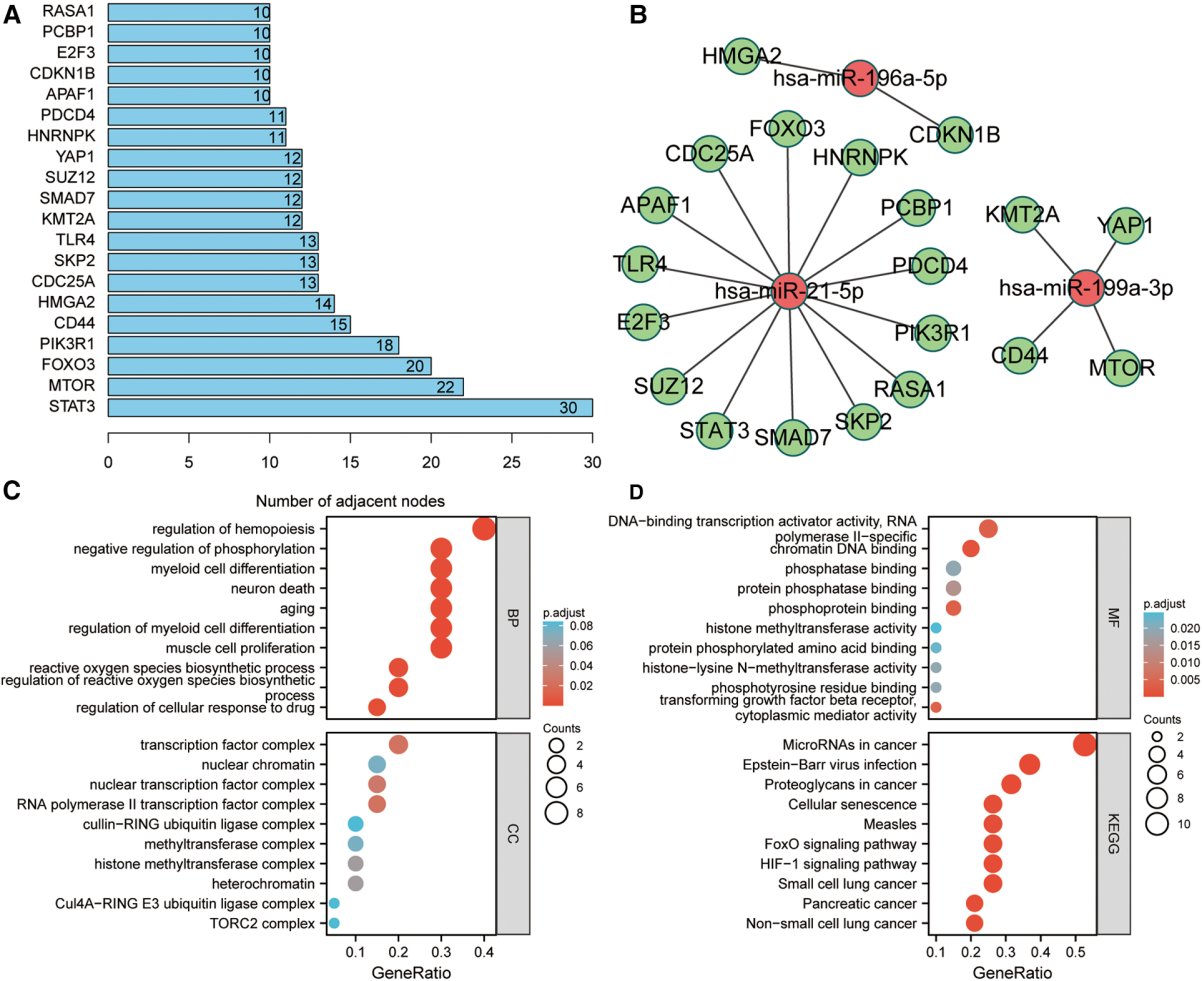
Identification of key target genes and miRNA–mRNA regulatory network construction. (**A**) Screening of top 20 genes in terms of molecular connectivity as key target genes as per the protein-protein interaction (PPI) network, which was constructed by STRING database. Each entry's numerical value indicates the level of connectivity among its genes, with higher values indicating greater connectivity. (**B**) Regulatory network of key target genes and miRNAs. The correlation between miRNA and gene suggests the presence of a regulatory association between the two entities. (**C,D**) GO and KEGG enrichment analyses of key target genes. The presented items of enrichment exhibit a adjust *P* < 0.05.

The PPI network of these potential target genes was constructed based on the STRING database, and the top 20 genes in terms of molecular connectivity were screened as the key genes (Figure 4A). Subsequently, a miRNA–mRNA regulatory network based on these 20 key genes and 4 miRNAs, was built (Figure 4B). GO enrichment analysis highlighted that these 20 key genes were also linked with the regulation of neuron death, neuron death, and aging (Figure 4C). KEGG enrichment analysis indicated that these 20 key genes were connected to several carcinogenesis-related pathways such as microRNAs in cancer, HIF-1 signaling pathway, proteoglycans in cancer, and pancreatic cancer (Figure 4D).

### 3.4. Identification of diagnostic biomarkers for DRE

**Figure 5:**
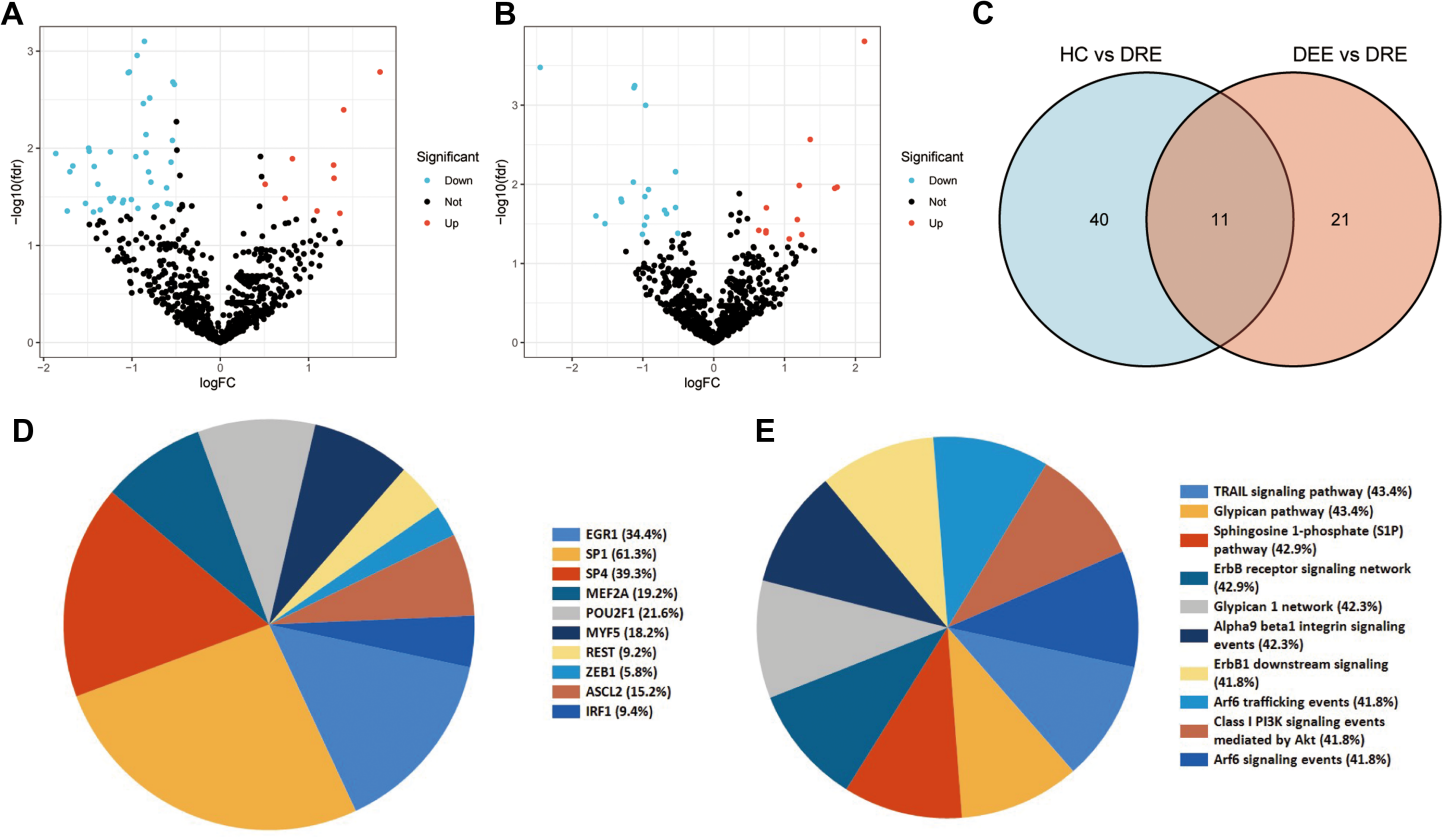
Identification and enrichment analysis of differential expression miRNAs (DEmiRNAs) in children with drug-resistant epilepsy (DRE). (**A**) Differential expression of plasma EV-derived miRNAs between children with DRE and healthy controls (HC). (**B**) Differential expression of plasma EV-derived miRNAs between children with DRE and children with drug-effective epilepsy (DEE). The blue dots denote miRNAs that have undergone down-regulation, while the red dots indicate miRNAs that have undergone up-regulation. (**C**) Identification of DEmiRNAs in children with DRE. Venn diagrams depict the intersections of differentially expressed miRNAs between two distinct groups. (**D**) Enrichment analysis of transcription factors (TFs) for differentially expressed miRNAs. (**E**) Pathway enrichment analysis of DEmiRNAs.

**Figure 6:**
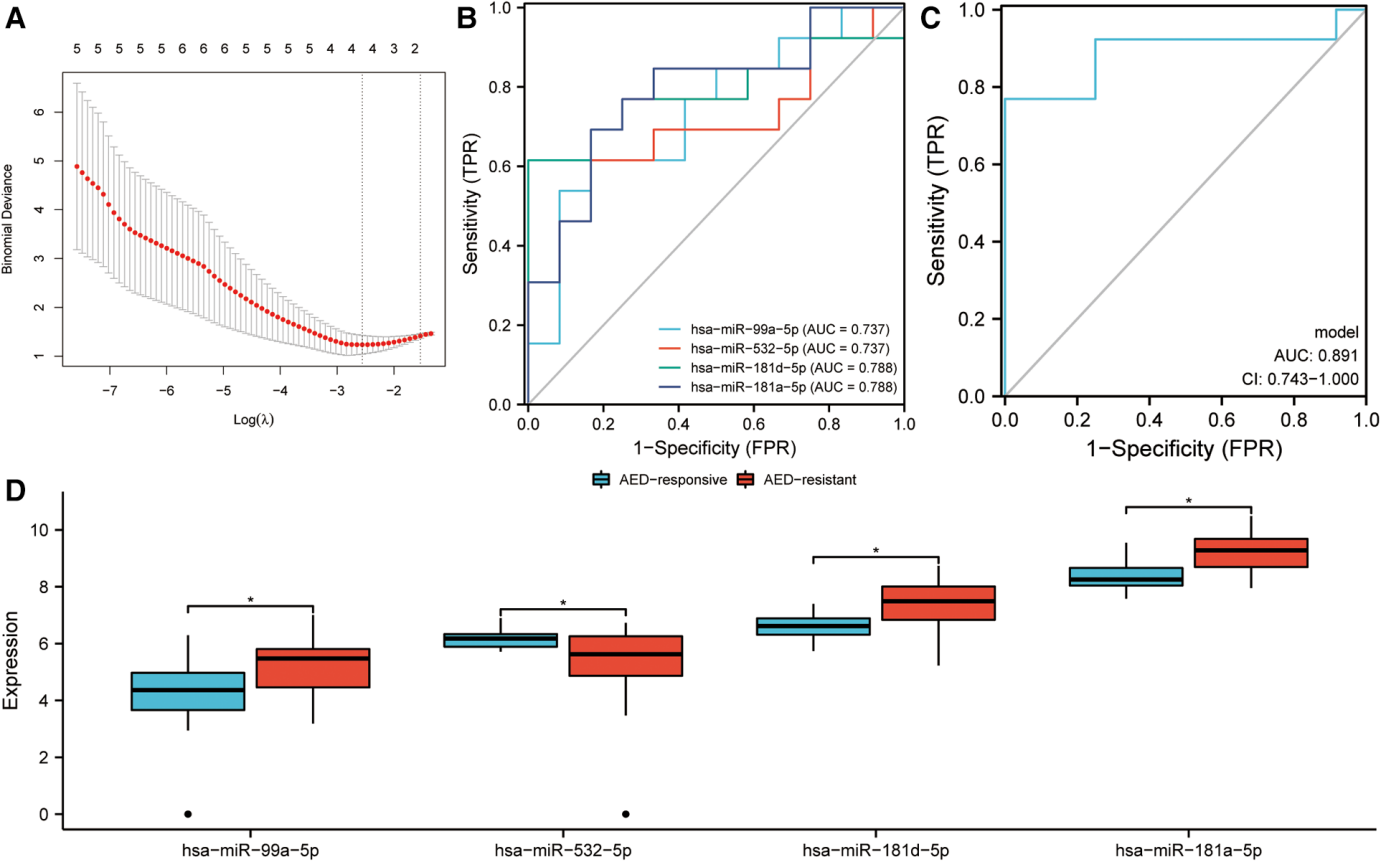
Identification of diagnostic markers in children with drug-resistant epilepsy (DRE). (**A**) LASSO regression was used to screen potential candidate miRNAs. (**B**) ROC curves assessing the diagnostic potential of the miRNAs. (**C**) ROC curves assessing the diagnostic efficiency of the diagnostic model, which was developed through logistic regression. CI, confidence interval. (**D**) Expression of four miRNAs in children with DRE and drug-effective epilepsy (DEE).

Early identification of DRE in children is essential in developing precise treatment plans and improving prognosis, thereby reducing the emotional and financial burden on families and society. In this study, 42 DEmiRNAs between children with DRE and normal healthy controls were identified. Among the 42 DEmiRNAs, nine miRNAs were aberrantly highly expressed and 33 miRNAs were down-regulated in children with DRE (Figure 5A). In addition, DEmiRNAs in EVs between children with DRE and those with DEE were analyzed, of which 12 miRNAs were upregulated and 20 were downregulated in children with DRE (Figure 5B). Subsequently, the two sets of results were intersected to identify specifically expressed miRNAs (SEmiRNAs) in children with DRE, yielding 11 miRNAs (Figure 5C). TF enrichment analysis revealed that the major transcription factors regulating these 11 miRNAs were EGR1, SP1, SP4, MEF2A, POU2F1, MYF5, REST, ZEB1, ASCL2, and IRF1 (Figure 5D). The pathway enrichment analysis proposed that these miRNAs were primarily linked with the activation-related pathways, including the TRAIL signaling pathway, Glypican pathway, and PI3K signaling events mediated by Akt (Figure 5E). Afterward, LASSO regression was used to further screen potential miRNA markers capable of distinguishing between children with DRE and those with DEE, yielding four miRNAs with diagnostic potential (Figure 6A). ROC analysis suggested that these four miRNAs, including hsa-miR-99a-5p, hsa-miR-532-5p, hsa-miR- 181d-5p, and hsa-miR-181a-5p performed well in differentiating children with DRE from those with DEE, with AUC values of 0.737 (0.534–0.940), 0.737 (0.523–0.952), 0.788 (0.592–0.985), and 0.788 (0.603–0.974), respectively (Figure 6B). A diagnostic model according to these four miRNAs was built, using logistic regression and the precise and comprehensive parameters are presented in Supplementary Table S2. ROC analysis showed a good diagnostic efficiency of this model, with an AUC value of 0.891 (0.743–1.00) (Figure 6C). Differential analysis suggested that hsa-miR-99a-5p, hsa-miR-181d-5p, and hsa-miR-181a-5p were significantly upregulated in plasma exosomes in children with DRE, whereas hsa-miR-532-5p was upregulated in children with DEE (Figure 6D).

**Figure 7:**
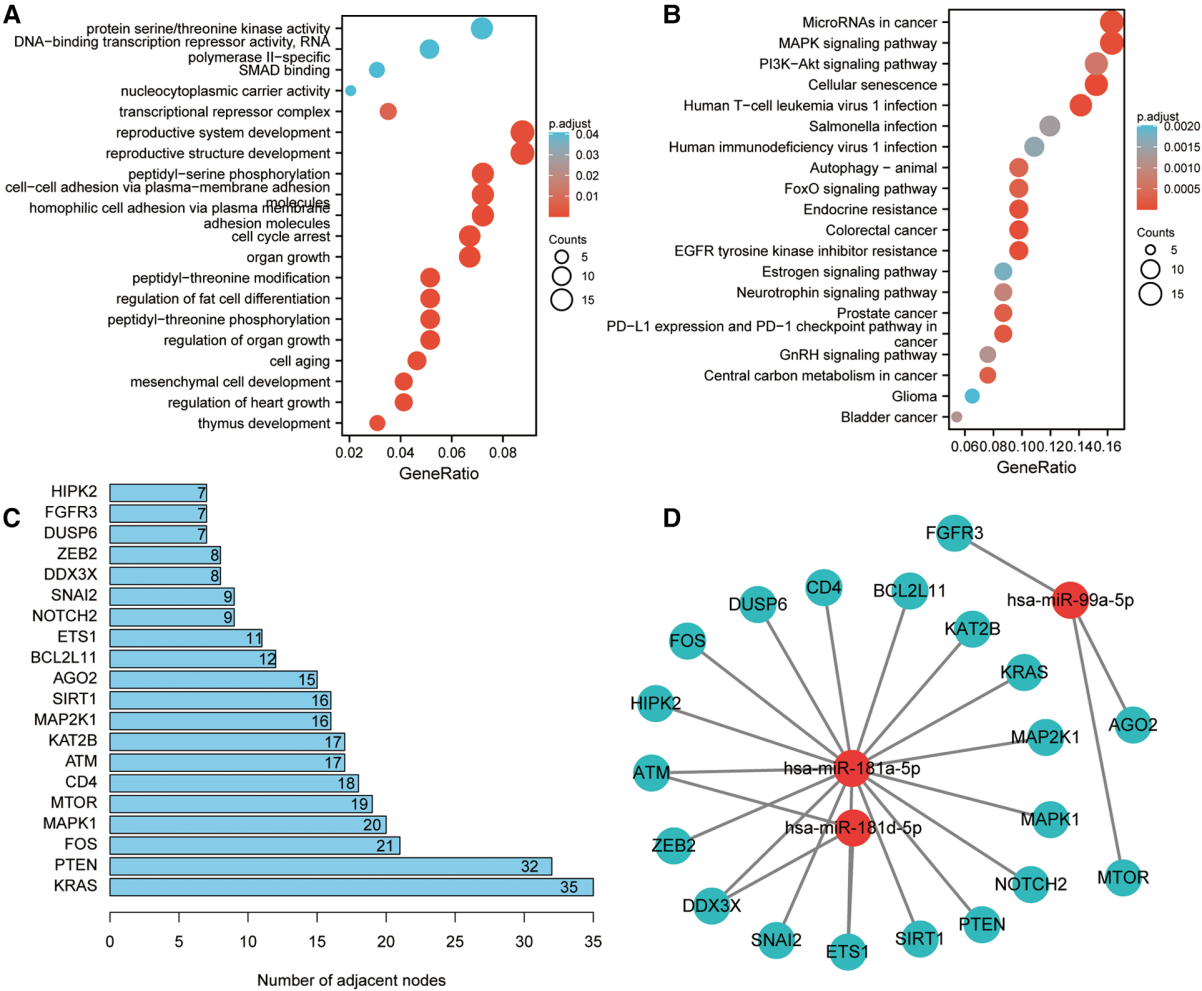
Target gene enrichment analysis and miRNA regulatory network construction. (**A**) GO enrichment analysis of target genes. (**B**) KEGG enrichment analysis of target genes. (**C**) Screening of top 20 genes in terms of molecular connectivity as key target genes as per the protein-protein interaction (PPI) network, which was constructed by STRING database. Each entry's numerical value indicates the level of connectivity among its genes, with higher values indicating greater connectivity. (**D**) Regulatory network of key target genes and miRNAs. The correlation between miRNA and gene suggests the presence of a regulatory association between the two entities.

The three miRNA target gene prediction websites: miRTarBase, TargetScan, and miRDB were utilized for the prediction of the potential target genes of these four miRNAs. It was suggested by KEGG enrichment analysis that the target genes were primarily enriched in microRNAs in cancer, MAPK signaling pathway, autophagy, endocrine resistance, and EGFR tyrosine kinase inhibitor resistance (Figure 7B). Subsequently, the PPI network was built and the first 20 genes ranked by molecular connectivity were screened as key genes (Figure 7C). The constructed miRNA–mRNA network showed potential regulatory relationships between hsa-miR-181-5p and multiple genes (Figure 7D).

## 4. Discussion

Although clinical manifestations and imaging data are frequently employed in the diagnosis and treatment of epilepsy, and are typically effective for patients who respond to medication, the absence of validated laboratory diagnostic markers for childhood epilepsy or drug-resistant epilepsy is a notable limitation. miRNA, a new biomarker, has exhibited strong clinical application potential in both early diagnosis and prognostic assessment of diseases. MicroRNAs have demonstrated potential as diagnostic or prognostic indicators in a range of chronic pediatric illnesses (34). The dysregulation of miRNA expression can be a significant contributor to the onset of seizures and neuronal death in epilepsy. Additionally, the identification of hsa-miR-146a-5p, hsa-miR-138-5p, and hsa-miR-187-3p as potential diagnostic markers for childhood epileptic encephalopathy holds promise for clinical applications (35). There was remarkable downregulation of miR-15a-5p and miR-194-5p in plasma of children with epilepsy, with high specificity and sensitivity in the early diagnosis of PE (36, 37). In addition, functional analysis revealed that miR-15a-5p and miR-194-5p have a vital function in the proliferation and apoptosis of hippocampal neuronal cells. Furthermore, plasma miR-9a-3p and miR-106b-5p have been extensively studied as markers in patients with epilepsy (38–41). EV, a unique pool of biomarkers, is rich in miRNAs and can protect miRNAs from degradation. Therefore, EV-derived miRNAs are good candidates for disease markers, and EVs in plasma can be used as markers for specific diagnoses of CNS diseases. In addition, miRNAs have higher stability in vesicles than mRNAs. Therefore, miRNAs in EVs may serve as diagnostic and prognostic markers for neurological disease (42–44). Results of a study suggested that miRNA biomarkers derived from plasma EVs may serve as viable diagnostic indicators for both childhood epilepsy and drug-resistant epilepsy (33).

This study analyzed the plasma exosome-derived miRNA expression profiles of children with epilepsy and normal healthy controls. In contrast to prior studies (33), our study employed a standardized approach to normalize TPM data using the SVA and limma package and screened for differentially expressed genes based on FDR values and *P*-values. This comprehensive analysis of miRNA expression profiles allowed for the identification of potential miRNA markers in children with epilepsy and drug-resistant epilepsy from various perspectives. Additionally, we utilized the LASSO regression technique to identify miRNAs with diagnostic potential in a rigorous and scientific manner. In addition, we selected miRNAs with relatively high expression abundance as candidate markers in our study. This methodology takes into account the intricacy of the statistical model and exclusively chooses the most pertinent variables. This approach enabled us to isolate miRNAs that exhibited both statistical disparities and physiological significance in epilepsy. Moreover, we anticipated the target genes of the distinct miRNAs and established regulatory networks utilizing various databases, which facilitated the exploration of the potential functions of these miRNAs. Additionally, we conducted an analysis of transcription factor enrichment to elucidate the upstream regulatory factors of the miRNAs, thereby facilitating the identification of potential causes for their differential expression. Our approach was characterized by a higher degree of specificity and comprehensiveness, enabling us to identify miRNAs that were not only statistically significant but also physiologically relevant. In total, 51 DEmiRNAs in children with epilepsy were screened based on differential analysis. TF enrichment analysis highlighted that SP1 and SP4 were considerably enriched for transcripts involved in regulating these miRNAs. In addition, SP1 and SP4 are involved in regulating epileptogenesis and could be potential therapeutic targets (45–48). In this study, four miRNAs (miR-1307-3p, miR-196a-5p, miR-199a-3p, and miR-21-5p) that can distinguish epileptic children from normal children were identified. Research by Johnson et al. suggested that the reduced expression of miR-1307-3p in the saliva of children who experienced concussion predicted a significantly high probability of conversion to persistent concussion (49). Li et al. reported that miR-199a-3p is dysregulated in neurons of children with epilepsy, which is regulated by lncRNA-TUBG1 and associated with neuron apoptosis and biological behaviors (50). Moreover, miR-21-5p has been implicated in a variety of neurological disease, i.e., epilepsy, dementia, Alzheimer's disease, and autism, along with regulating apoptosis of hippocampal neurons (17, 51–53). It has been suggested by the findings that these four miRNAs may be crucially involved in epileptogenesis. The diagnostic model constructed on these four miRNAs showed good potential in differentiating children with epilepsy, with an AUC value of 0.94. Three miRNA target gene prediction databases were incorporated for the prediction of the potential target genes of these four miRNAs and their potential functions. KEGG enrichment analysis of the target genes revealed their main involvement in the PI3K-Akt signaling pathway, MAPK signaling pathway, and cell cycle, all of which have been confirmed to be involved in epileptogenesis by several studies (54–58).

Approximately 1/3 of patients with epilepsy eventually develop DRE (59). Currently, both early diagnosis and treatment of DRE are unsatisfactory. In this research, four miRNAs (miR-99a-5p, miR-532-5p, miR-181d-5p, and miR-181a-5p) that could be used to diagnose DRE were identified based on LASSO regression. A diagnostic model incorporating these four miRNAs had an AUC value of 0.891. miR-99a-5p in cerebrospinal fluid was confirmed to be a marker of neurological disease in a study by Yoon et al. (60). The involvement of miR-532-5p in the onset and progression of Alzheimer's disease and its neuroprotective role in post-ischemic stroke had been suggested by several research studies (61–63). MiR-532 was also found to be significantly dysregulated in serum within a time frame of 200 min following a solitary seizure (64). In addition, miR-181a-5p is associated with the onset of several neurological disease and can be involved in the induction of seizures in epileptic mice (65, 66). The researchers also detected dysregulated expression of miR-181a in the hippocampus and plasma of patients with epilepsy, which may be involved in epileptogenesis by regulating the inflammatory response and may serve as a potential diagnostic marker for epilepsy (65, 67–73). It has been revealed by these results that the mentioned miRNAs have a role in the onset and progression of both epilepsy and DRE.

However, there are still some limitations to this study. We used a relatively small sample size for the present study. The findings from this study require confirmation by a larger prospective cohort study. It is worth noting that the sample size used for the model is consistent across various experimental groups, which may not be representative of real-world scenarios where the likelihood of having a child with epilepsy is lower. Furthermore, the absence of a distinct test dataset raises the possibility that the model may have exhibited overfitting tendencies. In addition, this study was a comprehensive bioinformatics study based on published data. The GEO data does not provide insight into whether the DEE or DRE groups exhibited varying frequencies of structural and/or genetic epilepsies. The absence of information regarding the specific type of epilepsy diagnosed in the patients may introduce bias into the results. Therefore, further *in vivo* and *in vitro* experiments are needed to validate this result in the future.

## 5. Conclusion

In conclusion, this research presented the miRNA expression profiles in characteristic plasma EVs among children with epilepsy and DRE by bioinformatics approaches. Overall, this study gives a novel understanding of the onset mechanism of epilepsy and DRE.

## Data Availability

The original contributions presented in the study are included in the article/**Supplementary Material**, further inquiries can be directed to the corresponding author.
